# Relationship between cognitive performance and mobility in patients with Parkinson’s disease: A cross-sectional study

**DOI:** 10.1590/1980-57642018dn13-040006

**Published:** 2019

**Authors:** Nariana Mattos Figueiredo Sousa, Roberta Correa Macedo

**Affiliations:** 1 Rede SARAH de Hospitais de Reabilitação Ringgold standard institution - Reabilitação Neurológica, Salvador, BA, Brazil.

**Keywords:** Parkinson’s disease, gait parameters, cognition, therapeutic strategies, doença de Parkinson, parâmetros da marcha, cognição, estratégias terapêuticas

## Abstract

Gait disorders may be associated with cognitive impairment, and slow speed predicts cognitive impairment and dementia. Objective: To investigate the relationships between cognitive function and gait performance in patients with Parkinson’s disease (PD) who attended a hospital neurorehabilitation program. Methods: Descriptive and inferential statistics (Pearson’s correlation) were used for data analysis. The cognitive functions were evaluated through Digit Span, Mental Control, Trail Making Test, Phonemic Verbal Fluency Task, and Addenbrooke’s Cognitive Examination III. The motor function was assessed through 10-meter walk test, Mini BESTest and Timed Up and Go Test. Results: A total of 65 patients were included in this study. Of these, 66.15% were males, mean age was 61.14 (8.39) years, mean educational was 12 (8) years, disease progression time was 5.45 (4.37) years. 64.61% were in stages I and II of the Hoehn and Yahr stage. The correlation analyses showed that balance skills are significantly correlated with the ability to switch attention between two tasks and visuospatial function. The function mobility showed a significant correlation with cognitive tests. Conclusion: Data suggest the importance of the aspects of switch attention and mental flexibility in gait, evidencing the greater difficulty for double tasks.

## RESUMO

Alterações na marcha podem estar associadas ao comprometimento cognitivo, e a velocidade lenta prediz comprometimento cognitivo e demência. Objetivo: Investigar as relações entre a capacidade cognitiva e o desempenho da marcha em pacientes com doença de Parkinson (DP) que participaram de um programa de neurorreabilitação hospitalar. Métodos: A estatística descritiva e inferencial (correlação de Pearson) foi utilizada para análise dos dados. As funções cognitivas foram avaliadas por meio do Teste de Extensão de Dígitos, Controle Mental, Teste de Trilha, Tarefa de Fluência Verbal Fonêmica, Exame Cognitivo de Addenbrooke III. A função motora foi avaliada através do teste de caminhada de 10 metros, Mini BESTest e *Timed Up and Go Test.* Resultados: Um total de 65 pacientes foi incluído neste estudo. Destes, 66,15% eram do sexo masculino, com média de idade de 61,14 (8,39) anos, com escolaridade média de 12 (8) anos, tempo de evolução da doença de 5,45 (4,37) anos. 64,61% estavam nos estágios I e II do estágio Hoehn e Yahr. As análises de correlação mostraram que as habilidades de equilíbrio estão significativamente correlacionadas com a capacidade de alternar a atenção entre duas tarefas e a função visuoespacial. A função mobilidade apresentou correlação significativa com os testes cognitivos. Conclusão: Os dados sugerem a importância dos aspectos de troca de atenção e flexibilidade mental na marcha, evidenciando a maior dificuldade para tarefas duplas.

Parkinson’s disease (PD) is a progressive neurodegenerative condition characterized by symptoms: tremor, stiffness and bradykinesia. Postural instability and gait impairment are also common symptoms. Non-motor manifestations such as disturbances of the autonomic nervous system, sleep disorders, depression and cognitive and neuropsychiatric disorders can either precede motor symptoms or appear throughout the course of the disease.^1^^,^ 2

Cross-sectional and longitudinal studies^3^^,^ 4 report that gait changes may be associated with cognitive impairment, especially executive functions. Reduction in gait velocity predicts cognitive impairment and dementia.^5^ Cross-sectional studies show an association between decline in mental flexibility skills and inhibitory control with reduced walking speed and falls risk, as well as divided attention deficit and difficulty gait initiation.^6^^,^ 7

Total scores for the Montreal Cognitive Assessment (MOCA) were positively correlated with total scores from motor tests such as Purdue pegboard test and Timed up and Go test (TUG), with worse motor scores associated with worse cognitive performance.^8^ There was also a significant association between cognitive impairment and postural instability gait difficulty subtype (PIGD) whereas there was no strong correlation with the tremor-dominant subtype.^9^^-^ 11

Patients with PD have limited ability to cope with complex tasks that require cognitive demands, ie perceiving and deflecting obstacles, dividing attention while keeping walking.^12^ The ability to adequately monitor and plan gait while dealing with cognitive overload is impaired in this population,^13^ as well as when performing dual tasks.^14^^,^ 15 The executive function deficit was associated with gait impairment, freezing of gait (FOG).^11^^,^ 16

Cognitive changes may be risk factors for poor gait performance and falls, what impact on patient’s community participation and independence. Therefore, it is important to conduct a study with larger samples, to further investigate the interaction between motor-cognitive variables. The purpose of the current study was to examine the relationship between motor and cognitive symptoms, determining which motor parameters are most strongly associated with cognitive parameters in PD patients.

## METHODS

This was a cross-sectional study. Patients with a PD diagnosis according to the criteria of the UK Brain Bank were recruited.^17^ These patients had attended the neurological rehabilitation inpatient or outpatient program at the SARAH Network of Rehabilitation Hospitals (Brazil). Inclusion criteria were: age greater than 50 years, educational level greater than 4 years, no psychiatric disorders prior to the diagnosis of PD, as well as no history of substance use and abuse; without behavioral, motor and/or sensory alterations that may interfere during the execution of the cognitive tests (all participants were ON stage of medication during the evaluation). We excluded patients with moderate or severe depressive symptoms (Beck Depression Inventory-BDI ≥20),^18^ Hoehn and Yahr Scale (H&Y) score IV and patients with dementia, in accordance with the Movement Disorder Society (MDS) PD-MCI Level II diagnostic criteria (2012).^19^

All subjects gave their written informed consent before entering the study, approved by the local ethics committee. 

### Cognitive assessment

Global cognitive and executive functions were assessed by tests validated for the Brazilian population, except the ACE-III, which is under validation process for the DP patients.

• Digit Span Test (forward and backwards) (WMS-R): It evaluated the storage capacity and reverberation in immediate verbal memory (phonological loop) and the ability to maintain and manipulate information (central executive), respectively.^20^

• Mental Control (WMS-R): It assessed mind control, attention and concentration.^20^

• Trail Making Test (TMT-A and B): It assessed motor speed, inhibitory control and mental flexibility.^21^

• Phonemic Verbal Fluency: It assessed inhibitory control (executive functions) and verbal fluency, which depends on an active search of words in the lexicon.^21^

• Global Cognition (ACE-III): It assessed five cognitive domains (attention, memory, verbal fluency, visuospatial ability, and language). The total score is 100.^22^

### Motor assessment

• 10-Meter Walk Test (10MWT): It assesses walking speed in meters per second over a 10-meter distance. Subjects were evaluated while walking in a comfortable speed.^23^

• Mini-BESTest: It evaluates dynamic balance through 14 tests (anticipatory postural adjustments, reactive postural control, sensory orientation, gait and dynamic balance. These tests are graded on a 3-level ordinal scale (0-2), according to the performance obtained, thus reaching a maximum score of 28 points in case of normal performance in all tests.^24^

• Timed Up and Go: The TUG test assesses mobility and balance. During cognitive TUG, the individual performs the same test, however performing a second motor task or cognitive task concomitantly. Patients were asked to perform the TUG associated with calculus tasks asked by the examiner.^25^

### Data analysis

Descriptive statistics were performed to characterize demographic and clinical data, inferential statistics through Spearman’s correlation coefficient was used to assess the association between gait parameters (velocity, balance and functional mobility) and cognitive test scores (single and global domain).

The variables (age, duration and severity of the disease) could have interfered in these results. To minimize these factors, multiple linear regression analysis was performed.

A p-value less than 0.05 was taken to indicate a statistically significant relationship between the corresponding pair of variables interrogated. All analyses were performed using Statistical Package for the Social Sciences (SPSS), version 22.0.

## RESULTS

Table 1 shows the demographic and clinical characteristics of the sample population (N=65). This group of DP patients had relatively short duration of disease (average 5.45±4.37 years), and had neither dementia (based MDS criteria), not moderate or severe depression (based BDI scores). The highest proportion was of male and in stages I-II of the H&Y scale, that is, there were not patients with significant postural instability. 

**Table 1:** Characteristics of the PD sample.

N= 65		Mean (SD)
Age		61.14 (8.39)
Gender: n (%)		66.15% (Male), 33.84% (Female)
Schooling (years)		12.44 (8.95)
PD duration (years)		5.45 (4.37)
Hoehn & Yahr Stage	I-II: n (%)	64.61%
	III-IV: n (%)	35.38% (III)
BDI		9.70 (4.34)

SD: Standard Deviation; BDI: Beck Depression Inventory.

The ACE-III subscores were lower than the expected for a healthy population, especially for attention, memory, fluency and visuospatial abilities (Attention/Orientation 16.05±1.84, Memory 19.69±4.52, Fluency 10.18±2.65, Language 25.15±2.03, Visuospatial 14.38±2.58).

In the other neuropsychological tests, it was observed a greater impairment in measures of working memory, mental flexibility and speed of mental processing (Digit Span Forward 5.72±5.66, Digit Span Backward 3.58±1.01, TMT-A second 70.30± 30.10, TMT-A mistake 2.47±1.12, TMT-B second 196.53± 101.23, TMT-B mistake 4.02±1.49, Phonemic Verbal Fluency 31.27±11.46).

Regarding the motor tests, the patients presented greater difficulty in the functional mobility test, mainly in the TUG-Cognitive. Whereas there was no risk of falls and/or impairment in the community walking (T10M-104.17±24.48; MiniBest-22.31± 40.09; TUG 0 or 1=48, 2=17).

For inferential statistics, there was a significant correlation between TUG scores and total score and all cognitive domains of ACE-III, as well as Mini-BESTest performance and visuospatial domains of ACE-III. It was also observed a significative correlation between performance on TUG and neuropsychological tests, such as mental control, trail A and B (time), digit span (forward and backward) and motor-H&Y scores. No associations were detected between any of the cognitive performance measures and 10-meter walk test (Table 2).

**Table 2:** Correlation between cognitive and motor variables.

Variables	Mini-BESTest	TUG	10MWT (cm/s)
Total score (ACE-III)	rSp =0,2072 (p=0.1291)	0.4839 (0.0002**)	0.0289 (0.834)
Attention/Orientation (ACE-III)	rSp =0.1824 (p=0.1459)	0.4416 (0.0002**)	-0.0354 (0.7792)
Memory (ACE-III)	rSp =0,2378 (p=0.0564)	0.3937 (0.0012**)	0.2102 (0.0929)
Fluency (ACE-III)	rSp =0.1759 (p=0.1611)	0.4173 (0.0005**)	0.0476 (0.7067)
Language (ACE-III)	rSp =0.1513 (p=0.229)	0.3308 (0.0071**)	0.0634 (0.6161)
Visuospatial (ACE-III)	rSp =0.2508 (p=0.0439*)	0.3846 (0.0016**)	0.054 (0.6695)
Mental Control (WMS)	rSp =0.2731 (p=0.0278*)	-0.2448 (0.0494*)	-0.01 (0.9367)
FAS (total)	rSp =0.0198 (p=0.9075)	0.2545 (0.1285)	-0.1276 (0.4517)
TMT-A (seconds)	rSp =0.2233 (p=0.0761)	-0.4215 (0.0005**)	-0.0376 (0.7681)
TMT-A (mistakes)	rSp = -0.1043 (p=0.4122)	-0.0391 (0.7593)	-0.0435 (0.7328)
TMT-B (seconds)	rSp = -0.2677 (p=0.0325*)	-0.4474 (0.0002**)	-0.0957 (0.4521)
TMT-B (mistakes)	rSp = -0.4474 (p=0.0002**)	-0.2194 (0.0841)	0.0125 (0.9227)
Digit Span (forward)	rSp =0.1988 (p=0.1123)	0.3665 (0.0027**)	-0.1219 (0.3332)
Digit Span (backward)	rSp =0.1634 (p=0.1933)	0.3837 (0.0016**)	-0.0009 (0.9431)
BDI	rSp = -0.2667 (p=0.0513)	-0.4106 (0.002**)	-0.112 (0.42)
H&Y	rSp = -0.4210 (p=0.0005**)	-0.3717 (0.0025**)	-0.2348 (0.0618)

ACE-III: Addenbrooke Cognitive Examination; FAS: Phonological Verbal Fluency; TMT: Trail Making Test; H&Y: Hoehn & Yahr Stage; BDI: Beck Depression Inventory; TUG: Timed Up and Go Test; 10MWT: 10-Meter Walk Test. rSp: Spearman's correlation coefficients.

* P-value < .05 (two-tailed).

** P-value < .001 (two-tailed).

As presented in the table 3, the result of the regression analysis showed that the ACE-III total score is associated to the cognitive TUG poor performance and the severity of the disease (measured by H&Y) is associated with the Mini-BESTest result.

**Table 3:** Multiple linear regression analysis.

Dependent Variables		ACE-III (Total)	Digit Span (forward)	Digit Span (backward)	TMT-A (seconds)	TMT-B (seconds)	Disease duration	H&Y
**TUG**	β	0.026	0.013	0.037	-0.003	0.001	0.006	-0.164
	p	0.057	0.360	0.728	0.364	0.632	0.762	0.277
**MiniBest**	β	-0.009	0.116	-0.249	-0.006	-0.003	-0.017	-2.315
	p	0.924	0.287	0.752	0.830	0.756	0.899	0.041*
**T10M**	β	-0.042	0.491	0.917	0.036	0.005	-0.477	-10.618
	p	0.944	0.452	0.847	0.828	0.928	0.565	0.117

ACE-III: Addenbrooke Cognitive Examination; TMT: Trail Making Test; H&Y: Hoehn & Yahr Stage; TUG: Timed Up and Go Test; T10M: 10-Meter Walk Test

* P-value < .05 (two-tailed).

Stratification of the sample was performed, since aspects such as age, duration and severity of the disease may interfere in the relationship between gait and cognition. As indicated in the table 4 the results were maintained when correlated with the functional mobility test. The TUG test showed a strong positive correlation with several tests, regardless of the stratification of its sample.

**Table 4:** Subgroup. Timed up Go Test.

TUG	<60a	≥60a	<5a (illness)	≥5a (illness)	H&Y I-II	H&Y III-IV
ACE-III (Total Score)	0.3598 (0.0773)	0.576 (0.0009**)	0.4577 (0.0143*)	0.5734 (0.0018**)	0.3934 (0.0176*)	0.5472 (0.0188*)
Attention/Orientation	0.2143 (0.2388)	0.6639 (0.007**)	0.4007 (0.0154*)	0.6054 (0.0005**)	0.3462 (0.0198*)	0.4983 (0.0299*)
Memory	0.3126 (0.0816)	0.4456 (0.0094**)	0.3632 (0.0294)	0.5216 (0.0037**)	0.2476 (0.101)	0.6349 (0.0035**)
Fluency	0.3732 (0.0354*)	0.4547 (0.0079**)	0.4877 (0.0025**)	0.4177 (0.0242*)	0.4429 (0.0023**)	0.3401 (0.1542)
Language	0.2253 (0.215)	0.4299 (0.0125*)	0.2662 (0.1166)	0.3456 (0.0663)	0.3296 (0.027*)	0.2564 (0.2894)
Visuo-spacial	0.3108 (0.0834)	0.4185 (0.0154*)	0.4694 (0.0039**)	0.2636 (0.1671)	0.371 (0.0121*)	0.3581 (0.1322)
Mental Control	0.1304 (0.4769)	0.3652 (0.0366*)	0.3687 (0.0269*)	0.1629 (0.3985)	0.1775 (0.2435)	0.4239 (0.0705)
FAS (total)	0.2774 (0.2983)	0.2963 (0.1922)	0.1331 (0.5985)	0.4678 (0.0434*)	0.2309 (0.289)	0.1348 (0.6459)
TRAIL-A (sec)	-0.4887 (0.0045**)	-0.3678 (0.0384*)	-0.4158 (0.013*)	-0.3178 (0.093)	-0.4365 (0.0027**)	-0.364 (0.1376)
TRAIL-B (sec)	-0.385 (0.0296*)	-0.4936 (0.0041**)	-0.4667 (0.0047**)	-0.3634 (0.0527)	-0.448 (0.002**)	-0.3993 (0.1007)
TRAIL-B (mistake)	-0.3338 (0.0619)	-0.128 (0.4926)	-0.4093 (0.0162*)	0.0286 (0.8827)	-0.217 (0.1572)	-0.2007 (0.4246)
Digit Span (forward)	0.2507 (0.1663)	0.4931 (0.0036**)	0.1895 (0.2683)	0.6271 (0.0003**)	0.2018 (0.1836)	0.5724 (0.0104*)
Digit Span (backward)	0.3597 (0.0432*)	0.4181 (0.0155*)	0.265 (0.1183)	0.5118 (0.0045**)	0.232 (0.1252)	0.5202 (0.0224*)

ACE-III: Addenbrooke Cognitive Examination; FAS: Phonological Verbal Fluency; TMT: Trail Making Test; H&Y: Hoehn & Yahr Stage; BDI: Beck Depression Inventory; TUG: Timed Up and Go Test.

* P-value < .01 (two-tailed).

** P-value < .05 (two-tailed).

## DISCUSSION

Gait is a complex task that requires integration of several domains, among them the cognitive functions. People with PD are able to improve their gait when they direct their attention to their walking movement, suggesting an impairment in the automatic control of the gait. Cognitive decline, especially of executive functions, is associated with gait disorders and risk of falls.^13^ Studies have shown the relationship between cognitive and motor skills, especially executive functions and bradykinesia in individuals with PD.^3^^,^ 15 The balance skills are significantly correlated with the ability to switch attention between two tasks.^22^ However, few studies have investigated these aspects through a cognitive assessment with patients in the early stage of disease (stage I-III of the H&Y scale). 

The study conducted by Wild et al. (2013) investigate the effect of dual-tasking on cognitive performance and gait parameters in patients with idiopathic PD, compared with healthy elderly control. The study found that individuals with PD have cognitive performance impacted by walking, indicating that this population prioritizes motor task over cognitive task.^26^

The current study identified that motor parameters were significantly associated with cognitive skills, especially aspects related to switch attention, evidencing that the balance ability is significantly correlated with the ability to allocate attention to tasks that are performed at the same time as found in similar research.^14^^,^ 15 This interaction has been identified in cognitive tests that assess inhibitory control (TMT-B), operational memory (digit span forward) and functional mobility test (cognitive-TUG). Patients with worse performance in functional mobility were those with greater cognitive impairment, suggesting of these motor symptoms are related to increased cognitive dysfunction.

Performing a cognitive task with a motor test has been adopted as a more sensitive predictor of fall, simulating real-world demands, where individuals usually are performing a mobility activity switching attention to another stimulus. Cognitive-TUG is an example of dual-task test which is recommended as part of a neurological screening for fall-risk.^27^ A retrospective cohort study conducted with people with PD investigated the benefit of adding a task (manual or cognitive) to TUG to identify fall risk in this population concluded that the insertion of a cognitive task to the TUG test is more accurate to detect fall risk in people with PD.^28^ In this way, it is possible to notice the role played by the attention abilities in the mobility, mainly in the community, where the multiple stimuli will compete with the motor task, potentially limiting the independence of those who have the attention impairment.

These results highlight the usefulness of attention evaluation in people with PD, as well as the importance of conducting the rehabilitation program to provide specific fall prevention guidelines and interventions when attention impairment is presented.

Although previous studies have reported this correlation between cognition and motor skills in Parkinson’s disease, few authors have conducted in-depth investigations of the relationship between cognitive deficits and motor dysfunctions involving gait balance and gait ability in PD patients. This present study not only a brief battery of cognitive evaluation was adopted, but also tests of attention and executive function were applied.^29^^-^ 31

This piece of research found no correlation between gait velocity (evaluated through the 10-meter walk test) and performance in cognitive tests. Perhaps studies that evaluate other gait parameters, such as step length, double support time or postural reactions, can more specifically detect how cognition impacts gait performance.

The present study had the following strengths: 1. Neuropsychological evaluation with appropriate cognitive measures; 2. Patients with homogeneous cognitive characteristics, without dementia; 3. Assessment performed on the ON stage of medication; 4. Balance and Mobility assessment through a quantitative tool. We point out the following limitations: 1. Small sample size; 2. Absence of evaluation of temporospatial gait data other than speed, especially cadence and simple and double support time.

The results of this study showed a significant correlation between motor and cognitive variables, mainly in attention measurement, mental flexibility and operational memory with functional mobility motor test (TUG). These data suggest the importance of divided attention aspects and mental flexibility in gait, suggesting a greater difficulty when two attentionally demanding tasks are performed simultaneously. 

This result indicates the need to work on these aspects throughout the rehabilitation program with activities involving divided attention, such as dual tasks training that may involves two motor activities or a motor activity performed while a cognitive task is done, as well as neuropsychological intervention (individual or group), with attention and executive skills training. In addition, patients and their caregivers should receive appropriate guidance and fall prevention counselling at home and in the community. 

These results have some clinical implications since they can help the identification of cognitive deficit and its correlation with motor impairments in patients with PD, contributing to adjuvant treatment through neuropsychological intervention and risk prevention methods for this population. Therefore, it’s important to explore the role of attention associated with therapeutic strategies to improve the gait in subjects with PD. In addition, we recommend further studies for greater evidence of this results.
